# US News and World Report Cancer Hospital Rankings: Do They Reflect Measures of Research Productivity?

**DOI:** 10.1371/journal.pone.0107803

**Published:** 2014-09-23

**Authors:** Vinay Prasad, Jeffrey A. Goldstein

**Affiliations:** 1 Medical Oncology Branch, National Cancer Institute, National Institutes of Health, Bethesda, MD, United States of America; 2 Prtizker School of Medicine, University of Chicago, Chicago, IL, United States of America; State University of New York, Oswego, United States of America

## Abstract

**Context:**

Prior research has faulted the US News and World Report hospital specialty rankings for excessive reliance on reputation, a subjective measure of a hospital's performance.

**Objective:**

To determine whether and to what extent reputation correlates with objective measures of research productivity among cancer hospitals.

**Design:**

A retrospective observational study.

**Setting:**

Automated search of NIH Reporter, BioEntrez, BioMedline and Clinicaltrials.gov databases.

**Participants:**

The 50 highest ranked cancer hospitals in 2013's US News and World Report Rankings.

**Exposure:**

We ascertained the number of NCI funded grants, and the cumulative funds received by each cancer center. Additionally, we identified the number of phase I, phase II, and phase III studies published and indexed in MEDLINE, and registered at clinicaltrials.gov. All counts were over the preceding 5 years. For published articles, we summed the impact factor of the journals in which they appeared. Trials were attributed to centers on the basis of the affiliation of the lead author or study principal investigator.

**Main Outcome:**

Correlation coefficients from simple and multiple linear regressions for measures of research productivity and a center's reputation.

**Results:**

All measures of research productivity demonstrated robust correlation with reputation (mean r-squared  = 0.65, median r-squared = 0.68, minimum r-squared = .41, maximum r-squared = 0.80). A multivariable model showed that 93% of the variation in reputation is explained by objective measures.

**Conclusion:**

Contrary to prior criticism, the majority of reputation, used in US News and World Rankings, can be explained by objective measures of research productivity among cancer hospitals.

## Background and Significance

Each year, the US News and World Report (US N&WR) ranks the 50 highest scoring US cancer centers as a part of its annual hospital specialty rankings. These rankings generate attention and criticism from the public, policy researchers, and physicians [1]–[3]. Although the rankings for 12 of 16 specialties (including cancer) are based on a combination of factors: reputation among specialists, survival statistics, patient safety data, nursing staffing information, nursing magnet status, and patient volume, the final scores have been faulted for excessively relying on reputation, a subjective element [4]–[6]. Prior research has found that the reputation score alone, based on a survey of specialists, most strongly correlated with the overall score for both adult [4] and pediatric [5] rankings. Additionally, among adult specialties, ranking on reputation alone agreed with final rankings 100% of the time [4]. Because reputation is ascertained via an opinion poll [7], the US N&WR rankings have been criticized for failing to provide an objective measure. [4]

**Figure 1:**
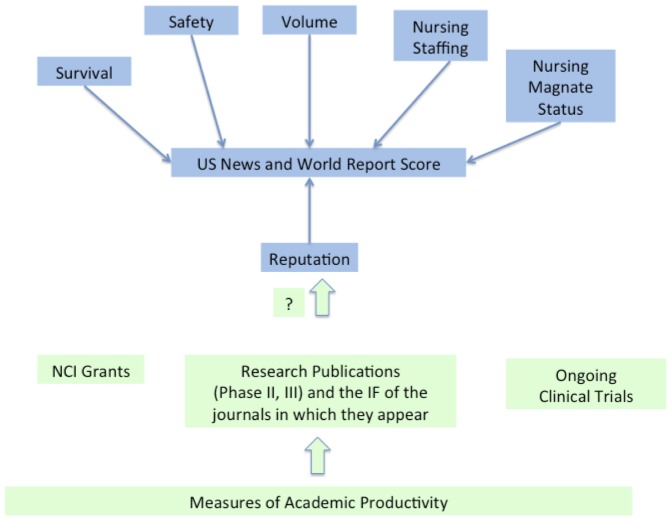
Diagram depicting the US News and World Report Score derivation, and our research question: whether measures of academic productivity correlate with the score.

We sought to assess to what extent reputation correlates with objective measures of academic or research productivity among cancer hospitals. Specifically, we assembled data pertinent to 3 measures of a center's research excellence: (1) Grant Data—the cumulative number and cumulative funds for National Cancer Institute (NCI) funded grants over the preceding 5 year period (2) Trial publication data—the number of Phase I, II and III trials with correspondence directed to a cancer center over the last 5 years; and, the cumulative impact factor of journals in which these trials appeared, and (3) Completed and ongoing trials—the number of Phase I, II and III trials listed for each center in clinicaltrials.gov. Phase I trials are those that test the tolerability of a novel agent or novel drug combination at varying doses. Phase II trials measure the activity of a novel drug or combination, and may use a prospective control arm or historical benchmark, and Phase III trials are those designed to formally assess the efficacy of a drug against an acceptable alternative standard of care. We sought to assess what proportion of the variability in reputation could be explained by these 3 objective measures of research performance. We considered the last 5 years to provide a contemporary sample of each center's performance in these metrics. **Figure 1** depicts our hypothesis graphically.

## Methods

### US News rankings

We collected information from the US News and World Report Annual Hospital Rankings in the last published year at the time of our investigation (2013). Data used to generate the rank and final score were extracted. Specifically, this included information on cancer center reputation, survival, patient safety, nursing staffing and nursing magnet status. A full description of the US News and World Report methodology is available here: http://health.usnews.com/health-news/best-hospitals/articles/2013/07/16/how-we-ranked-the-best-hospitals-2013-14-an-faq.

### National Cancer Institute Sponsored Grants

NCI grant data were obtained in the following manner. A copy of the NIH ExPORTER database was downloaded (Date: 12/2/2013). Grants funded by the National Cancer Institute (NCI) with start dates between 2008 and 2013 were considered. The institution given for each grant was matched with a ranked institution in automated fashion using Python. We generated simplified institution names, removing punctuation, articles, prepositions, and institution types, such that “Cleveland Clinic, Lerner College of Medicine” was simplified to “Cleveland Lerner”. For institutions known by multiple names, e.g. The University of Washington Cancer Center and/or The Fred Hutchinson Cancer Center, separate matching was performed for each name and the resulting grant numbers and amounts were combined. All grants were manually checked (by JAG) to ensure accurate attribution to a cancer center. There were a small number of grants with total payments of 0 or 1 dollar. We considered these grants as trivial and removed them from final totals.

### Published clinical trials and cumulative impact factor

Automated searches were performed using BioEntrez and BioMedline on (Date: 4/28/2014) using Python packages and the Pubmed syntax. Searches were performed for publications dated between 2008 and 2013 using the major MeSH subject heading: cancer. Publication type [PT] was specified as Phase I, Phase II or Phase III, and multiple permutations were used for center names, such as “Dana-Farber” or “Brigham and Women's”, etc. To increase return, we also searched all publications, omitting the publication type and instead using the key words Phase I, Phase II or Phase III added. Impact factors for each publication were calculated by querying the 2012 impact factors provided by http://www.citefactor.org/impact-factor-list-2012.html. Because of differences in the abbreviation systems used, not all papers were successfully paired with a journal impact factor. For phase I publications, 4866/5973 (81%) trials were paired. For phase II publications, 1343/1580 (85%) were paired and, among phase III trials, 411/437 (94%) were paired.

### Clinical trials listed at Clinicaltrial.gov

We searched for all trials at clinicaltrials.gov with the search term ‘cancer’, yielding in 39,422 studies. These trials downloaded in XML format (4/28/2014), and provided data on the trial, start date, study id, phase of drug development, source of funding and lead institution. We considered trials with start dates between 2008-2013. Trials were collated based on the source institution and merged with institution names. Because single institutions may be known under several names, the merged list of 2702 institutions was manually searched using fragments of the institution name e.g. ‘Cleveland’ and ‘Lerner’ for the Cleveland Clinic Lerner College of Medicine.

### Statistical Analysis

Statistical analysis was performed using Stata Version 13 (Stata Corp, College Station, TX). Descriptive statistics for the data are provided. Simple linear and multiple linear regressions were used.

## Results

We analyzed data for the 50 highest ranked US News and World Report Cancer Hospitals. In addition to the 6 variables used to derive each center's final score, we assembled data on the number of, and total payments for NCI funded grants, the number of phase I, II and III publications, the cumulative impact factor of the journals in which those publications appeared, and the number of clinical trials listed by center and phase in clinicaltrials.gov, all over the last 5 years. A summary of variables used by the USN&WR and the measures of research productivity appear in **Table 1**.

**Table 1:** Characteristics of the top 50 Cancer Hospitals, as ranked by the US News and World Report.

	Descriptive data of the Top 50 Cancer Centers (Range and Percentile)	Low	25th percentile	Median	75th percentile	High
US News and World Report Data	Final Score	53.1	55.6	60.8	65.9	100
	Reputation; Percent of Respondents Naming It a “Top Center”	0	2.1	4	7	67.7
	Survival*	8	9	9	10	10
	Safety*	1	1	2	2	3
	Volume (discharges)	169	1203	1660	2195	5529
	Nursing Staffing*	1.3	1.9	2.2	2.4	3.6
	Magnet Status (0 or1)	0	1	1	1	1
Measures of Research Productivity	Total Grant Funding (dollars)	0	60 million	150 million	320 million	850 million
	Number of Grants	0	184	393	678	1477
	# of Phase I Trials (published)	0	4	13	24	91
	# of Phase II Trials (published)	0	7	23	44	135
	# of Phase III Trials (published)	0	2	5	10	41
	Impact Factor of Published Phase I trials	0	27	84	163	734
	Impact Factor of published Phase II trials	0	72	145	270	1242
	Impact Factor of published Phase III trials	0	23	64	123	616
	# of Phase I trials (Clinicaltrials.gov)	0	9	20	35	181
	# of Phase II Trials (Clinicaltrials.gov)	0	10	21	33	210
	# of Phase III Trials (Clinicaltrials.gov)	0	0	1	3	26

*Standardized units.

**Figure 2:**
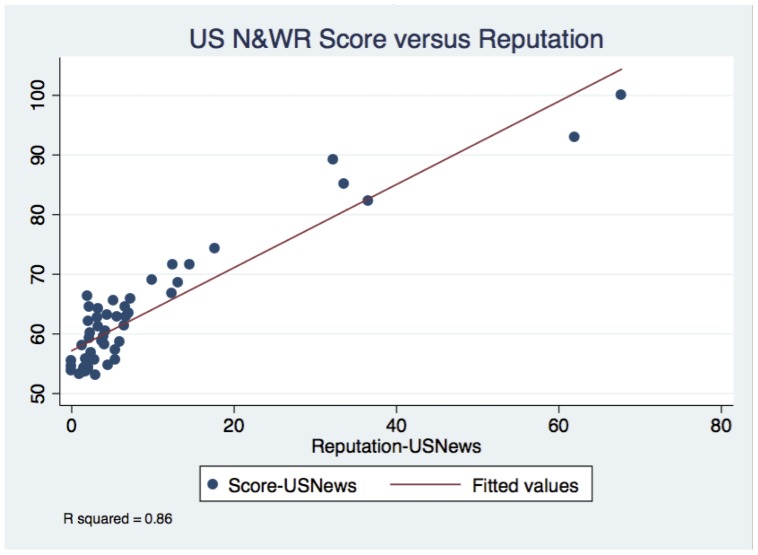
The relationship between US News and World Report Score and Reputation.

We first sought to verify the results of prior reports. We asked: what percentage of variability in score is explained by each of the 6 variables used by the US News and World Report to construct their final score. The results of a multiple linear regression testing this question are shown in **Table 2**. All 6 predictors reached statistical significance, and, the overall r-squared was 0.94. Thus, 94% of the variation in final score could be explained by these predictors. Simple linear regressions for each of the 6 variables against the final score are shown in **Table 3**. Only reputation (r-squared  = 0.86) and patient volume (R∧2 = 0.48) had correlation coefficients greater than 0.1 when tested individually. **Figure 2** graphically depicts the relationship between the final score and reputation, the variable with strongest correlation.

**Table 2:** A multiple linear regression of the US News and World Report Score and its contributors.

	Beta Coefficient	95% CI (low-high)		P Value
Reputation; Percent of respondents who named the center as a “Top Hospital”	0.585	0.503	0.666	<0.001
Survival*	2.514	1.308	3.719	0.00013
Safety*	2.945	1.796	4.093	0.00001
Volume (discharges)	0.001	0	0.002	0.02914
Nursing Staffing*	2.441	0.561	4.32	0.01216
Magnet Status (0 or1)	3.22	1.314	5.127	0.00144

*Standardized units.

r-squared  = 0.94.

**Table 3:** Correlation coefficients (R2) from simple linear regressions between the US News and World Report Score and its contributors.

	Reputation	Survival	Safety	Volume	Nursing Staffing	Nursing Magnet Status
US News Score	0.86	0.08	0.08	0.48	0.03	0.01

We then explored our hypothesis: whether and to what extent objective measures of research output correlate with reputation. Our hypothesis is depicted graphically in Figure 1. **Table 4** shows correlation and beta coefficients between the measures of research productivity we developed and the US News and World Report cancer hospital reputation score. All measures of research productivity demonstrated correlation with reputation (mean r-squared  = 0.65, median r-squared  = 0.68, minimum r-squared  = 0.41, maximum r-squared  = 0.80). Additionally, there was strong co-linearity among the variables, demonstrated by strong pair-wise correlation coefficients between all variables (**Table S1**). A multiple linear regression using all of our predictors against reputation yielded an overall r-squared of 0.93 **(Full model details in Table S2).**

**Table 4:** Correlation coefficients (R2) between US News and World Report Reputation Score, and measures of research productivity.

Simple linear regressions to predict reputation					
	Correlation Coefficient (R∧2)	Beta Coefficient	95% CI (low-high)		P value
Total Grant Funding	0.45	4.7E–8	3.2E–8	6.2E–8	<0.001
Number of Grants	0.41	0.0251	0.0163	0.0339	<0.001
# of Phase I Trials (published)	0.65	0.564	0.4443	0.6836	<0.001
# of Phase II Trials (published)	0.72	0.3789	0.3106	0.4472	<0.001
# of Phase III Trials (published)	0.65	1.2644	0.9932	1.5356	<0.001
Impact Factor of Published Phase I trials	0.74	0.0756	0.0625	0.0886	<0.001
Impact Factor of published Phase II trials	0.8	0.0464	0.0397	0.0531	<0.001
Impact Factor of published Phase III trials	0.68	0.0827	0.0662	0.0993	<0.001
# of Phase I trials (Clinicaltrials.gov)	0.58	0.3961	0.299	0.4932	<0.001
# of Phase II Trials (Clinicaltrials.gov)	0.79	0.3697	0.3144	0.4251	<0.001
# of Phase III Trials (Clinicaltrials.gov)	0.69	2.7211	2.1889	3.2533	<0.001

## Discussion

The US News and World Report hospital specialty rankings are influential and contentious [1], [4]. Prior work has shown that the final score is largely driven by reputation rankings, a subjective measure of hospital quality [4]–[6]. Indeed, our analysis confirmed this fact. We found that 85% of the variation in final score was explained by reputation among cancer hospitals. Moreover only reputation and patient volume demonstrated correlation coefficients greater than 0.10 (Table 2).

We then sought to assess whether and to what extent reputation correlates with objective measures of research productivity. Specifically, whether NCI funded grants, clinical trial publications and their impact factors, and ongoing clinical trials reflected reputation (Table 1). Indeed, we found that each individual measure of research productivity exhibited a positive and robust correlation with reputation (Table 4).

Additionally, we found that nearly all the variation in reputation could be explained by a combination of our objective measures (r-squared  = 0.93). While the interpretation of each coefficient from this multiple linear regression is limited due to collinearity among the variables (Table S1), collinearity does not affect the correlation coefficient.

Our findings appear to differ from a prior investigation, which measured publication output in the field of urology, and found little correlation between USN&WR rankings and the authors' ranking [8]. That study however focused solely on correlations between final rankings, as opposed to correlations between a center's publication score and USN&WR final score. As such, correlations may have been missed due to a singular focus on rank.

In short, we confirmed that US News and World Report cancer hospital rankings are largely based on reputation. However, while the reputation survey administered by the US News and World report is subjective, the subjective impressions of specialists in each field appear to correlate strongly with the rate of grant funding and number of clinical trials conducted by each center. Subjective impressions appear to approximate objective differences.

### Limitations

There are several limitations to our analysis. We chose to assess the correlation between a subjective measure of a cancer hospital—its perceived reputation according to the US News and World report—against objective measures of research productivity. It must be acknowledged that research is just one part of what cancer hospitals do, and we were not able to consider the quality of care provided by cancer hospitals, nor were we able to consider basic science research that may identify novel therapeutic targets in cancer. For instance, we did not consider the number of high impact basic science publications, which may someday lead to novel drugs or targets produced by center. Nevertheless, Phase I, II and III clinical trials are widely considered an important measure of research productivity in oncology, and pathways for drug development along this track are mature and robust. As such, we acknowledge that our variables themselves may only approximate measures of cancer center success, which are difficult to fully capture.

For any particular cancer center, we may have made errors in both the inclusion and exclusion of grants, articles or trials. While we believe that any such errors of measurement are unlikely to result in a systematic bias, we must acknowledge this as a limit. Additionally, we relied on the impact factor of the journal in which a trial publication appeared. This however is an imperfect approximation of the impact of any paper, and any given trial may be more or less important than the impact factor of the journal in which it was published. Finally, although our results suggest that much of the variation of reputation can be explained through objective measures (93%), we did not find that all of the variation could be explained. Thus, there may still be a subjective component to reputation rankings. To what extent this exists and whether or not it is detrimental may still be debated. However, in general we feel that our analysis was performed based on a fair assessment of objective indices of a cancer center's research productivity.

Finally, our results should not be misinterpreted to imply that the US News and World Report cancer hospital rankings are valid or that they are useful. We did not assess whether centers that achieved higher rankings provide better care, nor did we perform a systematic review to assess whether the rankings provide useful information to consumers and policy experts. Instead, we selected a very focused question: whether a key component of a hospitals score: their reputation, developed via a survey, correlates with objective measures of research productivity. For this very focused question, we provide a favorable answer.

## Conclusion

Contrary to prior criticism, we found that the majority of variation in the US News and World Report reputation score can be explained by objective measures of research productivity among the top 50 ranked cancer hospitals. These results do not imply the USN&WR rankings are valid or useful.

## Supporting Information

Table S1 All pairwise correlation coefficients (R2) between US News and World Report Reputation Score, and measures of research productivity.
(DOCX)

Table S2 Multiple linear regression for all factors versus reputation.
(DOCX)
