# Supplementary material for: US News and World Report Cancer Hospital Rankings: Do They Reflect Measures of Research Productivity?
Source: PLoS One. 2014 Sep 23;9(9):e107803. doi: 10.1371/journal.pone.0107803 (PMC4172593; doi:10.1371/journal.pone.0107803)
Supplement: Table S1 — All pairwise correlation coefficients (R2) between US News and World Report Reputation Score, and measures of research productivity. (DOCX) [file pone.0107803.s001.docx]

|  | US News Reputation | Total Grant Funding | Number of Grants | # of Phase I Trials (published) | # of Phase II Trials (published) | # of Phase III Trials (published) | Impact Factor of Published Phase I trials | Impact Factor of published Phase II trials | Impact Factor of published Phase III trials | # of Phase I trials (Clinicaltrials.gov) | # of Phase II Trials (Clinicaltrials.gov) | # of Phase III Trials (Clinicaltrials.gov) |
| --- | --- | --- | --- | --- | --- | --- | --- | --- | --- | --- | --- | --- |
| US News Reputation | **~** |  |  |  |  |  |  |  |  |  |  |  |
| Total Grant Funding | **0.45** | ~ |  |  |  |  |  |  |  |  |  |  |
| Number of Grants | **0.41** | 0.95 | ~ |  |  |  |  |  |  |  |  |  |
| # of Phase I Trials (published) | **0.65** | 0.46 | 0.46 | ~ |  |  |  |  |  |  |  |  |
| # of Phase II Trials (published) | **0.72** | 0.52 | 0.49 | 0.88 | ~ |  |  |  |  |  |  |  |
| # of Phase III Trials (published) | **0.65** | 045 | 0.45 | 0.61 | 0.74 | ~ |  |  |  |  |  |  |
| Impact Factor of Published Phase I trials | **0.74** | 0.49 | 0.49 | 0.94 | 0.84 | 0.63 | ~ |  |  |  |  |  |
| Impact Factor of published Phase II trials | **0.80** | 0.51 | 0.47 | 0.83 | 0.96 | 0.78 | 0.86 | ~ |  |  |  |  |
| Impact Factor of published Phase III trials | **0.68** | 0.45 | 0.44 | 0.60 | 0.76 | 0.95 | 0.65 | 0.82 | ~ |  |  |  |
| # of Phase I trials (Clinicaltrials.gov) | **0.58** | 0.38 | 0.43 | 0.54 | 0.52 | 0.53 | 0.57 | 0.57 | 0.50 | ~ |  |  |
| # of Phase II Trials (Clinicaltrials.gov) | **0.79** | 0.47 | 0.46 | 0.62 | 0.62 | 0.58 | 0.68 | 0.70 | 0.61 | 0.89 | ~ |  |
| # of Phase III Trials (Clinicaltrials.gov) | **0.69** | 0.26 | 0.26 | 0.48 | 0.48 | 0.48 | 0.56 | 0.58 | 0.50 | 0.76 | 0.82 | ~ |

Table S1: All pairwise correlation coefficients (R2) between US News and World Report Reputation Score, and measures of research productivity.

|  |
| --- |
