# Supplementary material for: US News and World Report Cancer Hospital Rankings: Do They Reflect Measures of Research Productivity?
Source: PLoS One. 2014 Sep 23;9(9):e107803. doi: 10.1371/journal.pone.0107803 (PMC4172593; doi:10.1371/journal.pone.0107803)
Supplement: Table S2 — Multiple linear regression for all factors versus reputation. (DOCX) [file pone.0107803.s002.docx]

|  | | | | |
| --- | --- | --- | --- | --- |
|  | Beta Coefficient | 95% CI (low-high) | | P value |
| Total Grant Funding | -1.10E-09 | -3.57E-08 | 3.35E+08 | 0.94921 |
| Number of Grants | -0.001 | -0.0205 | 0.0184 | 0.91667 |
| # of Phase I Trials (published) | -0.46 | -0.8567 | -0.0549 | 0.02692 |
| # of Phase II Trials (published) | 0.15 | -0.1399 | 0.439 | 0.30221 |
| # of Phase III Trials (published) | 1.21 | 0.3954 | 2.0293 | 0.00469 |
| Impact Factor of Published Phase I trials | 0.06 | 0.0095 | 0.1036 | 0.01973 |
| Impact Factor of published Phase II trials | 0.01 | -0.0292 | 0.0421 | 0.71718 |
| Impact Factor of published Phase III trials | -0.08 | -0.1346 | -0.0183 | 0.01129 |
| # of Phase I trials (Clinicaltrials.gov) | -0.39 | -0.595 | -0.1938 | 0.0003 |
| # of Phase II Trials (Clinicaltrials.gov) | 0.47 | 0.2761 | 0.6695 | 0.00002 |
| # of Phase III Trials (Clinicaltrials.gov) | 0.43 | -0.3541 | 1.2108 | 0.27476 |

Table S2: Multiple linear regression for all factors versus reputation
